# Ground Reaction Forces during Vertical Hops Are Correlated with the Number of Supervised Physiotherapy Visits after Achilles Tendon Surgery

**DOI:** 10.3390/jcm10225299

**Published:** 2021-11-15

**Authors:** Łukasz Sikorski, Andrzej Czamara

**Affiliations:** 1 Department of Physiotherapy, College of Physiotherapy in Wrocław, 50-038 Wrocław, Poland; a.czamara@wsf.wroc.pl; 2 Center of Rehabilitation and Medical Education, 50-038 Wrocław, Poland

**Keywords:** physical therapy, rehabilitation, gastrocnemius muscle, ankle, Achilles tendon

## Abstract

The objective of this study was to assess the effectiveness of, and the correlation between, an average of 42 supervised physiotherapy (SVPh) visits for the vertical ground reaction forces component (vGRF) using ankle hops during two- and one-legged vertical hops (TLH and OLH, respectively), six months after the surgical suturing of the Achilles tendon using the open method (SSATOM) via Keesler’s technique. Hypothesis: Six months of supervised physiotherapy with a higher number of visits (SPHNVs) was positively correlated with higher vGRF values during TLH and OLH. Group I comprised male patients (*n* = 23) after SSATOM (SVPh x = 42 visits), and Group II comprised males (*n* = 23) without Achilles tendon injuries. In the study groups, vGRF was measured during TLH and OLH in the landing phase using two force plates. The vGRF was normalized to the body mass. The limb symmetry index (LSI) of vGRF values was calculated. The ranges of motion of the foot and circumferences of the ankle joint and shin were measured. Then, 10 m unassisted walking, the Thompson test, and pain were assessed. A parametric test for dependent and independent samples, ANOVA and Tukey’s test for between-group comparisons, and linear Pearson’s correlation coefficient calculations were performed. Group I revealed significantly lower vGRF values during TLH and OLH for the operated limb and LSI values compared with the right and left legs in Group II (*p* ≤ 0.001). A larger number of visits correlates with higher vGRF values for the operated limb during TLH (*r* = 0.503; *p* = 0.014) and OLH (*r* = 0.505; *p* = 0.014). An average of 42 SVPh visits in 6 months was insufficient to obtain similar values of relative vGRF and their LSI during TLH and OLH, but the hypothesis was confirmed that SPHNVs correlate with higher relative vGRF values during TLH and OLH in the landing phase.

## 1. Introduction

The contraction of the gastrocnemius and soleus muscles creates translational force through the Achilles tendon (AT), which results in plantar flexion of the foot. This function is very significant in propulsion while walking, running, and jumping [1]. As a result of overloads or excessive amounts of jumping, tendinitis, tendinopathy, and chronic, partial, or total rupture of the AT often occur, especially in the landing phase (eccentric contraction of triceps surae) [2,3,4]. The orthopedic examination includes anamnesis, the application of diagnostic tests, and ultrasound imaging to make individual diagnoses [5,6]. There is no uniform procedure concerning the surgical and conservative treatment of AT rupture [7,8]. In the case of complete rupture of the AT in physically active people who participate in sports activities with high dynamic movement and who have functional disorders, surgical treatment is recommended [9]. Clinical and functional outcomes following surgical repair of the AT are significantly improved compared with following conservative management [10]. Delayed repair of the AT compared with acute repairment should not make a difference in future AT rupture scale scores or the AT resting angle [11]. There are three main surgical methods for torn AT: open, percutaneous, and mini-open [12]. Rehabilitation treatment after AT rupture is essential for surgical treatment [13]. However, the importance of postoperative physiotherapy has not been taken into account [14,15]. Nevertheless, more attention has begun to be paid to the importance of early postoperative physiotherapy after surgical suturing of the AT [16]. Gould et al. (2021), in their systematic review, discovered and implied that detailed protocols were recommended for up to 12 weeks after surgery, which was most often continued as part of jogging or exercises performed in the gym [17]. The application of individual exercises at a given stage and in the longer period of postoperative physiotherapy after AT sewing has already been described [18,19]. Thus far, the issue of supervised or unsupervised physiotherapy has not been described after the use of AT suturing.

Supervised postoperative physiotherapy is carried out by a physiotherapist in a hospital or outpatient clinic based on a detailed protocol agreed with the physician. Most often, one visit lasts from 1.5 to 2 h in direct contact with a physiotherapist, with a high frequency of visits for at least 3 to 4 months for patients who want to return to everyday activity. For patients who want to return to physical activity, supervised physiotherapy should last at least 6–8 months. In addition, depending on the stage of postoperative physiotherapy, the patient has to perform the exercises recommended by a physiotherapist at home. Unsupervised postoperative physiotherapy is not fully explained here, as it is performed independently by a patient at home, who continues the recommended exercise procedure by themselves without the physiotherapist’s supervision. Partially supervised physiotherapy revers to when a patient rarely meets with a physiotherapist to discuss changes to the physiotherapy program regarding exercise methods, depending on the time that has passed since surgery. The use of unsupervised or partially supervised physiotherapy procedures is a cost-saving practice [20]. Applying an appropriate protocol of postoperative physiotherapy is just as important as choosing the right surgical treatment for a ruptured AT [21].

Supervised postoperative physiotherapy in patients, carried out by a physiotherapist, has already been described after anterior cruciate ligament (ACL) reconstruction in the literature [22]. The current protocol of supervised postoperative physiotherapy used for the patients after anterior cruciate ligament reconstruction significantly improves the values of jumping ability, muscle strength, and agility [23]. Glazebrook et al. (2019) emphasized the importance of patient supervision in the course of physiotherapy procedures after the nonsurgical treatment of acute AT rupture [24]. This problem is crucial, as we are still in search of the answer to the following question: when can a player or a patient, after surgical suturing of the AT, return to sports requiring dynamic locomotion activities, such as running, jumping, and hopping? Dams et al. (2019) indicated that the lack of a consistent postoperative physiotherapy protocol may be the reason for different recovery periods [25]. After the surgical suturing of AT, approximately 20% of patients do not regain their full athletic performance level preceding AT rupture [26]. Olson et al. (2011) noted that there was no significant improvement in jumping height between the first and second years following AT rupture in patients who had undergone nonsurgical or surgical treatment [27]. According to Willy et al. (2017), even 6 years after the surgical suturing of AT, compensations can be found in patients’ knee joints, especially during running and jumping activities [28]. Moreover, a premature return to sports after surgical suturing of the AT may contribute to disorders of the load-bearing axis in the knee joint, AT damage and overload, and even knee joint injuries [29]. Nilsson-Helander et al. (2010) assessed the return to physical activity after the surgical suturing of AT, based on drop counter-movement jump performance (drop CMJ) and hopping, by measuring their height and the time of contact between the feet and the surface [30].

It is difficult to find publications assessing conducted and supervised postoperative physiotherapy sessions, concerning the level of symmetry of the relative vertical ground reaction forces component during vertical hops in the landing phase after the surgical suturing of the AT, using an open method via Keesler’s technique. According to Wearing et al. (2020), one of the basic criteria for allowing athletes patients to return to sport after surgical suturing of the AT is the restoration of dynamic and symmetrical jumps [31].

The aim of this study was to assess the effectiveness of an average of 42 supervised postoperative physiotherapy visits carried out by a physiotherapist based on the mean of 6 highest vertical ground reaction force values obtained for two- and one-legged vertical hops after a 6 month physiotherapy program, and the limb symmetry index (LSI) in patients who aimed to return to physical activity after surgical suturing of the AT using Keesler’s technique.

It was hypothesized that the higher the number of supervised physiotherapy visits six months after the surgical suturing of the AT using Keesler’s technique, the higher the average of relative vertical ground reaction force values obtained by the patients will be.

## 2. Materials and Methods

The study was carried out at the Center of Rehabilitation and Medical Education and the College of Physiotherapy in Wroclaw, according to the guidelines of the Declaration of Helsinki. The participants and the Ethics Committee at the College of Physiotherapy in Wroclaw Resolution 1/2019, the Ethics Committee at the College of Physiotherapy in Wroclaw Resolution 1/2012, and the Senate Committee of The Ethics of Research at the Academy of Physical Education in Wroclaw 2006 provided written consent for the research. The study had a retrospective cohort study design and was conducted between 2006 and 2019.

### 2.1. Participants

Initially, 78 participants (males *n* = 69, females *n* = 9) were included in the study, who started and continued postoperative physiotherapeutic procedures after surgical suturing of the AT using an open method, in the Center of Rehabilitation and Medical Education physiotherapy where the study was conducted. All the patients attended the abovementioned center of their own free will, according to the recommendations of the operating physician, who recommended the rehabilitation protocol [19]. The following inclusion criteria were applied: complete rupture of the AT; male or female between 20 and 60 years of age, who were given treatment following unilateral surgical suturing of the AT using an open method only; absence of postoperative complications and concomitant diseases; implementation of conducted and supervised postoperative physiotherapy in one center for 6 months; activity level higher than 6 according to the Tegner Activity Scale (TAS) [32]. One type of AT suturing using Keesler’s technique was applied.

**Figure 1:**
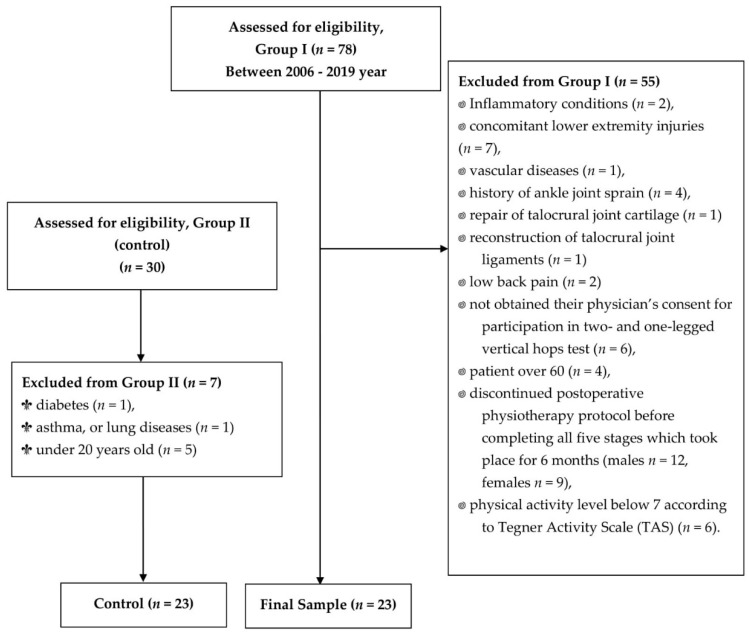
Flow diagram of the study; *n*—number of individuals.

Based on the anamnesis and medical documentation, some patients were excluded due to the following reasons: inflammatory conditions (*n* = 2); concomitant lower extremity injuries (*n* = 7); vascular diseases (*n* = 1); history of ankle joint sprain (*n* = 4); repair of talocrural joint cartilage (*n* = 1); reconstruction of talocrural joint ligaments (*n* = 1). Next, patients with lower back pain (*n* = 2), patients who had not obtained their physician’s consent for participation in two- and one-legged vertical hop tests (*n* = 6), patients over 60 (*n* = 4) and under 20 years of age (*n* = 0), those who discontinued the postoperative physiotherapy protocol before completing all 5 stages that took place over 6 months (males *n* = 12, females *n* = 9) [19], and patients who had a physical activity level below 7 according to the TAS (*n* = 6) [32] were excluded from the study. Due to the exclusion criteria, only males were included in Group I. In the beginning, 30 males without AT ruptures and level 7 physical activity according to the TAS were included in Group II (control). On the basis of anamnesis, subjects from Group II with diabetes (*n* = 1), asthma, or lung diseases (*n* = 1), and those under 20 years old (*n* = 5) were excluded. The algorithm for selecting subjects for the examined groups is presented in the flowchart of the study (Figure 1).

Finally, the participants that qualified for further analysis included 23 patients (males *n* = 23; females *n* = 0) after surgical suturing of the AT using Keesler’s technique, who were assigned to Group I. All patients, before AT rupture, participated in sport disciplines such as soccer, volleyball, and tennis. Group II included 23 males with no AT or talocrural joint injuries (Table 1). According to the TAS, the levels of physical activity for patients in Group I and Group II were 7 or higher [32]. Group I patients participated in an average of 42.22 visits (from 24 to 83 visits) as part of the 6 month supervised postoperative physiotherapy program after the surgical suturing of the AT. It was assumed that these patients participated in 42 visits (Table 1). It is worth mentioning that the physiotherapist had no impact on the number of visits, as this depended on the patients’ financial resources and commitment. The duration of one supervised physiotherapeutic session with a physiotherapist was 2 h. The studied groups were uniform in terms of age, but, statistically, they were significantly different in terms of body mass (*p* = 0.019) and height (*p* = 0.004). For this reason, the vertical ground reaction forces component values obtained for two- and one-legged vertical hops were normalized to body mass (relative ground reaction forces), expressed in N·kg^−1^. The comparison of patients’ body mass index (BMI) values did not reveal any statistically significant differences between the studied groups (*p* = 0.312). At every stage of physiotherapy, patients were additionally guided by the physiotherapist on how to perform exercises at home.

Group I included patients after unilateral AT continuity rupture surgical treatment by the open method using Keesler’s approach [33]. In Group I, the right leg was the dominant limb in 91.3% of patients (*n* = 21), and the left leg was the dominant limb in 8.69% (*n* = 2). A total of 47.82% of patients (*n* = 11) underwent surgery of the dominant leg. In Group II, the right leg was the dominant limb in 95.65% of patients (*n* = 22), while the left leg was the dominant limb in 4.34% (*n* = 1) of patients. Subsequently, the patients participated in the 6 months program of postoperative physiotherapy, carried out and supervised by a physiotherapist [19].

### 2.2. Postoperative Physiotherapy

In Group I, supervised postoperative physiotherapy was carried out, based on the protocol described in another publication [19]. This paper presents a simplified scheme of supervised postoperative physiotherapy. Initially, in the first stage, the patient was instructed on how to walk with two crutches after talocrural joint immobilization and non-weight-bearing exercises, and how to perform an isometric exercise and proprioceptive exercises.

Following the decision to remove immobilization during physiotherapy procedures, in the second stage of postoperative physiotherapy, cryotherapy was applied within approximately 3 min alongside isometric exercises, followed by an alternating magnetic field and laser therapy of postoperative scars. During the next sessions, the procedures were gradually prolonged, following standard recommendations accepted in physiotherapy practice. Further, the following exercises were applied: passive range of motion with CPM device, isometric exercise with gradual resistance, proprioceptive exercises, and walking in orthotics with two crutches. Whole-body stability and non-weight-bearing exercises were performed in low positions. The measurement of the vertical component (N) of ground reaction forces was taken using MTD Balance platforms during unconstrained double-leg standing, and then during single-leg standing on the uninvolved leg. The measurement of vertical ground reaction force values in each patient allowed an individual selection of load exerted on the operated lower limb for the vertical component during workout performance. The load was gradually increased every 3 days, ranging from 40 to 50 (N), provided that there was no pain or swelling in the area of the talocrural joint of the operated leg. Next, exercises were carried out on a cycle ergometer (initially with the orthosis). Therapeutic transverse massage was applied to areas including the triceps surae, soft tissues, the operated AT and ankle area, the postoperative scar, and the foot. Phonophoresis with an anti-inflammatory agent was also applied. The dose of phonophoresis was gradually increased under the standard methodology of the procedure. The plantar flexion angle was gradually reduced in the orthosis according to the surgeon’s recommendation. Lymph drainage and electrostimulation of the triceps surae were performed using a bipolar approach within 3–5 min.

Between the 7th and 12th weeks following surgery, the 3rd stage of postoperative physiotherapy started as follows: learning to walk without crutches was gradually introduced, the technique of performing individual phases of gait was improved, and the orthosis was permanently set aside. Then, the range of motion was increased in all planes of the operated tendon and the ankle joint. As in the previous stage, therapeutic massage was applied alongside first-degree mobilization of the triceps surae, the postoperative scar, the buttocks, the hindfoot, and the forefoot. Active and isometric exercises were introduced with gradually increased resistance of large muscle groups acting on the ankle joint with body stabilization and stretching exercises of the triceps surae and the hindfoot of the operated limb. The physiotherapist introduced exercises to improve neuromuscular coordination.

Between the 13th and the 16th postoperative weeks, the 4th stage of postoperative physiotherapy began as follows: exercises were performed to improve the strength of the muscles acting on the ankle joint and the entire operated and unoperated limbs, increasing the pressure exerted on the operated leg and also on the surface above body mass, as well as isometric, eccentric, and concentric–eccentric exercises with progressive resistance. Additionally, in the physiotherapy protocol, exercises were introduced to regain trotting, running, strength, and endurance abilities to prepare the patient for recreational activities.

Between the 17th and the 24th postoperative weeks, the 5th stage of postoperative physiotherapy began. Exercises were introduced to prepare the patient for the return to sport—these included vertical and countermovement jumping exercises; plyometric exercises, running at maximum speed, and changing movement directions; and specific exercises aimed at improving power, speed, and agility, adapted for a particular sport and the individual patient’s capabilities [19].

### 2.3. Clinical Trial

Prior to the measurement of vertical ground reaction force values, the participants in Groups I and II underwent an orthopedic assessment performed by a specialist physician. They were asked about the occurrence of pain measured using the 100 mm visual analogue scale (VAS) and a subjective sensation of ligament and ankle joint capsule stability. The physician conducted tests to assess the stability and continuity of the AT using Thompson’s test (Table 2). Moreover, the continuity of the abovementioned structures was assessed using ultrasound, and no pathology was revealed. The participants were asked about other injuries and operations of the lower limb and lower back.

### 2.4. Measurements of the Vertical Ground Reaction Forces Component

**Figure 2:**
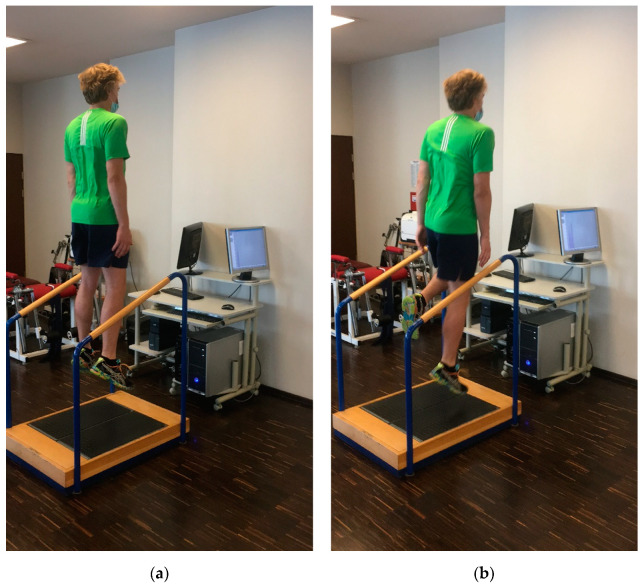
(**a**) Two-legged vertical hop; (**b**) one-legged vertical hop.

In both experimental groups, vertical ground reaction forces measurements were taken, and the values were expressed in newtons (N) using two force plates, namely, MTD Balance (MTD Systems, Neunburg v. Wald Germany) for the right and left lower legs, respectively. The measurements were based on the aforementioned methodology of the vertical ground reaction force value measurements [34]. The subjects wore sports clothing and shoes. Before the measurement, for 12 min, a warm-up was performed on a cycle ergometer with a constant velocity of 50–60 revolutions per minute (rpm) [34]. The warm-up was followed by a 5 min break. The force plates were adjusted to indicate 0 newtons (N) of vertical ground reaction force values, expressed before a patient stepped on them. Before the test, the subjects were instructed by the examiner on the techniques of two- and one-legged vertical hops. Two-legged vertical hops were presented to the subjects as two-foot ankle hops with primary motion at the ankle joint [35]. Participants were instructed to perform hopping with the highest possible height, with their hands placed along the torso. During the landing phase, the subject’s feet had to adhere to the ground with the toes and mid-foot (amortization) during the transition between landing and beginning the next vertical hops in the shortest possible time. Each vertical hop was performed in the upright position. The protocol did not allow arm movement or changing the direction of hopping (Figure 2a).

The subject had to perform one-legged vertical hops in the same way as two-legged vertical hops, but with the knee joint of the unexamined limb bent at 70°–90° during the test and in standing position (Figure 2b).

The main aim of the test was the measurement of the vertical ground reaction force values during the landing phase. Each participant performed a few trials of two- and one-legged vertical hops until they felt comfortable with the protocol.

Starting the actual test, at first, the vertical ground reaction force values during two-legged vertical hops were measured. The monitor screen was directed towards the examiner controlling the course of the measurement. At the beginning, the participant placed his right foot on the middle of the right force plate and his left foot on the middle of the left force plate. The examiner started the test and registration measurement using a computer program during two-legged standing. On the command “start”, the vertical ground reaction force values during two-legged vertical hops were measured consecutively in the landing phase—when participants performed at least 10 vertical hops correctly, an examiner used the verbal command “stop”. During a 10 s break, the subjects stood on the middle of both force plates to restore their balance and then proceeded to stand on one lower limb on the middle of the force plate. On the command “start“, the vertical ground reaction force values during one-legged vertical hops were measured consecutively in the landing phase—when participants performed at least 10 vertical hops correctly, the examiner used the command “stop”. Next, during a 10 s break, the subjects stood on the middle of both force plates to restore their balance and then proceeded to stand on the second limb on the middle of the force plate. On the command “start”, the vertical ground reaction force values during one-legged vertical hops were measured consecutively in the landing phase—when participants performed at least 10 vertical hops correctly, the examiner used the command “stop”. When the test was completed, the examiner saved the test in the computer program. One-legged vertical hops started with the non-operated followed by the operated leg. In Group II, the participants started the test with the right leg and continued with the left leg, according to the same methodology as that applied in Group I. The measurement was taken once in both groups.

We assumed that measurement could be interrupted when participants signaled that they were experiencing pain, fatigue, or were unable to maintain their hopping pace (slowing down or pausing on the platform). All the subjects from Groups I and II performed double- and single-leg vertical hop tests without experiencing the above-mentioned symptoms. For further assessment, the 6 highest vertical ground reaction force values were selected, measured during the contact between the foot and the surface in the landing phase. Then, the mean value was calculated from the 6 highest vertical ground reaction force values for each leg for double- and single-leg vertical hops during the landing phase [34]. Moreover, the mean vertical ground reaction force value was normalized for each patient’s body mass and expressed in N∙kg^−1^ (relative vertical ground reaction forces—RvGRF).

The measurement was taken by one examiner, as mentioned in previous studies with the same test conditions, based on the test–retest results in the intraclass coefficient (ICC) compartment, ranging from 0.84 to 0.95 [36]. To avoid bias, the measurement results were analyzed by an independent examiner. A similar article, concerning the measurement of vertical ground reaction forces during hopping, was described as an easy and reliable method for the assessment of the lower limb musculoskeletal function even in a small study population (6 male and 4 female) [37]. Based on the aforementioned studies, no power analysis of Group I was carried out.

### 2.5. Measurements of Circumferences of Ankle Joint and Shin and Range of Motion of the Foot

The ankle circumference was measured in the supine position at the level of the lateral and medial ankle. The shin circumference was measured 20 cm from the base of the patella. The active range of motion was measured with a goniometer. The initial position of each patient was supine in order to measure the range of plantar flexion and dorsiflexion of the foot. The axis of rotation of the goniometer was positioned below the lateral ankle [38]. The full range of motion for the sagittal plane was calculated by adding plantar flexion and dorsiflexion movement of the tested foot. Walking in a straight line was supervised by a physiotherapist, who assessed whether the gait was unassisted, alternating, and whether it met the 6 basics gait determinants [39]. The pain was assessed using a standard (100 mm) VAS scale. AT integrity was assessed with Thompson’s test performance [40].

### 2.6. Statistical Analysis

The number of individuals was indicated as *n*. The minimum size of the tested sample was not determined due to the retrospective and observational design of the study. Microsoft Office Excel 2007 (Microsoft, Redmond, WA, USA) and IBM SPSS 23 (IBM, Armonk, NY, USA) programs were used for the statistical analysis. The limb symmetry index (LSI) was calculated for each patient for two- and one-legged vertical hops by dividing the mean of the 6 highest vertical ground reaction force values (N) obtained for the operated leg by the mean of the 6 highest vertical ground reaction force values (N) obtained for the unoperated leg and multiplying the result by 100. For Group II, the LSI value was calculated by dividing the mean of the 6 highest vertical ground reaction forces (N) value obtained for the right leg by that obtained for the left leg, then multiplying the result by 100. Regarding limb symmetry assessment, values closer to 100 were used, and values below 90 or above 110 indicated asymmetry [36].

The analysis first calculated the mean values (x) and standard deviations (SD) for the particular standard features. The Shapiro–Wilk test was used to verify the distribution normality of the studied variables. The parametric t-test for independent samples was carried out for *p*-values > 0.050, obtained for both attempts, depending on the distribution normality. The Wilcoxon test was performed for *p*-values < 0.050 obtained from at least one attempt. One-way ANOVA was used for the comparison of relative vertical ground reaction force values obtained for the operated legs in Group I and for the right and left legs in Group II. When the significance level was at *p* < 0.050, Tukey’s post hoc test was applied. Independent *t*-tests were used for the between-group comparisons in Group I and Group II. Pearson’s linear correlation coefficient (*r*) was calculated for the force and direction of the linear correlation between the number of supervised postoperative physiotherapy visits and the relative vertical ground reaction forces during double- and single-leg vertical hops in Group I. The values corresponding to all two-dimensional associations were classified as negligible (0.00–0.30), low (0.31–0.50), moderate (0.51–0.70), high (0.71–0.90), and very high (0.901–1.00) [41]. The statistical significance level was set at *p* < 0.050.

## 3. Results

### 3.1. Comparison of Pain Assessment, Thompson’s Test Results, 10 m Unassisted Walking, Obtained Ankle Joint and Shin Circumferences, and Range of Motion (ROM) of the Foot in the Between-Limb and Between-Group Analyses

Participants from Group I and II reported no pain on the VAS scale, and Thompson’s test showed a normal AT integrity reflex. Additionally, 10 m unassisted walking was positively completed in both groups. There were no significant differences between the studied groups in the circumferences of shin and ankle joint and foot range of motion in the full range of motion in the sagittal plane, plantar flexion, and dorsiflexion of the foot. The between-limb comparison showed a significantly higher circumference of operated legs in Group I (*p* ≤ 0.001, Table 2). A significantly lower circumference of operated legs was found in Group I (*p* ≤ 0.001, Table 2). There were no significant differences between right and left legs in Group II, regarding the circumferences of the ankle joint and shin, or the range of motion of the foot (Table 2).

### 3.2. Comparison of Obtained Relative Vertical Ground Reaction Force during Two- and One-Legged Vertical Hops and Their LSI in the Between-Limb and Between-Group Analyses

**Figure 3:**
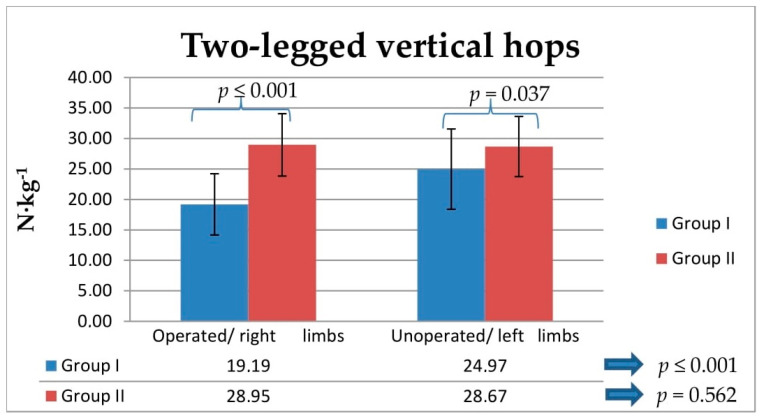
Comparison of the between-group and intragroup values of the vertical component of ground reaction forces during two-legged vertical hops, normalized for body mass, N∙kg^−1^, in Group I and Group II. Statistical significance level (*p*).

**Figure 4:**
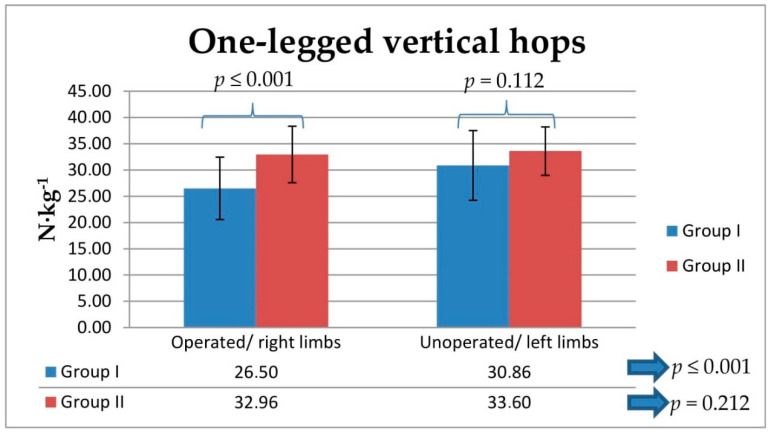
Comparison of the between-group and intragroup values of the vertical component of ground reaction forces during one-legged vertical hops, normalized for body mass, N∙kg^−1^, in Group I and Group II. Statistical significance level (*p*).

**Figure 5:**
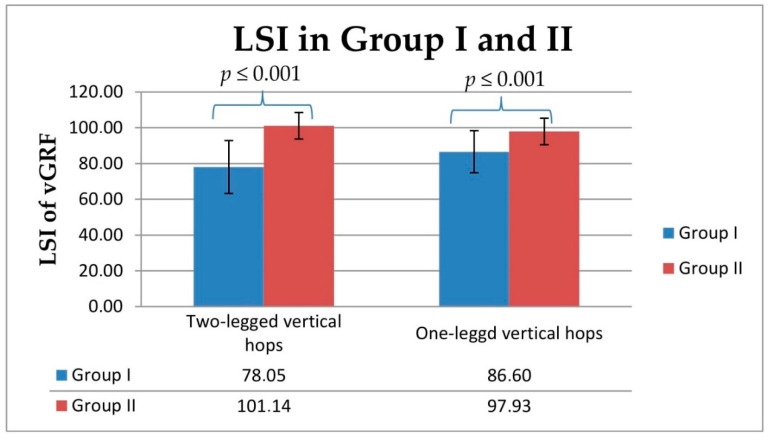
Comparison of the between-group limb symmetry index (LSI) values of the vertical component of ground reaction forces during two- and one-legged vertical hops in Group I and Group II. Statistical significance level (*p*).

The intragroup analysis showed statistically lower relative ground reaction force values during two- and one-legged vertical hops in Group I between operated and unoperated legs (*p* ≤ 0.001; Figure 3 and Figure 4). The between-group analysis showed statistically lower relative vertical ground reaction force values during two-legged vertical hops obtained for operated and unoperated legs in Group I compared with the values obtained in Group II for right and left legs (from *p* ≤ 0.001 to *p* = 0.037; Figure 3). The between-group analysis showed statistically lower relative vertical ground reaction force values during one-legged vertical hops obtained for operated legs in Group I compared with the values obtained in Group II for right legs (*p* ≤ 0.001; Figure 4). The LSI values obtained for Group I for two- and one-legged vertical hops were significantly lower for the lower extremities that had been operated on, compared with the unoperated side (*p* ≤ 0.001; Figure 5).

The between-group ANOVA analysis showed statistically lower relative vertical ground reaction force values obtained from the comparison between the values corresponding to operated legs in Group I and the values obtained in Group II for right and left legs during one- and two-legged vertical hops (*p* ≤ 0.001; Table 3). Tukey’s test revealed significantly lower relative vertical ground reaction force values for operated legs compared to those corresponding to right and left legs in Group II, obtained during one-legged vertical hops (*p* ≤ 0.001; Table 3) and two-legged vertical hops (*p* ≤ 0.001; Table 3). There were no significant differences between right and left legs in Group II in one-legged vertical hops (*p* = 0.913; Table 3) or two-legged vertical hops (*p* = 0.981 Table 3).

### 3.3. Association of Number of Supervised Postoperative Physiotherapy Visits with Relative Vertical Ground Reaction Forces and Limb Symmetry Index (LSI) of Vertical Ground Reaction Forces during Two- and One-Legged Vertical Hops

A significant and moderately positive correlation was found between the larger number of supervised postoperative physiotherapy visits and the higher relative vertical ground reaction force values, obtained during two-legged vertical hops for operated legs in Group I (*r* = 0.503; *p* = 0.014), as well as between a larger number of supervised postoperative physiotherapy visits and higher relative vertical ground reaction force values, obtained during one-legged vertical hops in Group I (*r* = 0.505; *p* = 0.014; Table 4).

## 4. Discussion

The authors, responding to the purpose of the study, conducted an average of 42 supervised postoperative physiotherapy visits in 6 months after AT surgery in Group I. This was insufficient to obtain similar values of average relative vertical ground reaction forces and their LSI in operated limbs, to those obtained from non-operated limbs and to those of the results obtained in the Group II control during two- and one-legged vertical hops.

Our research showed that the hypothesis—that a higher number of supervised physiotherapy visits carried out in six months after surgical suturing of the AT would result in higher values of the average vertical ground reaction forces during two- and one-legged vertical hops—was confirmed.

The functional tests, such as Thompson’s test and 10 m unassisted walking, did not show any differences between the two studied groups. The range of motion in the sagittal plane and the circumference of the ankle joint and shin did not differ between groups.

The authors aim to inspire other researchers to conduct further study in the future to answer the following question: what is the impact of more frequent visits supervised and conducted by a physiotherapist, according to one physiotherapeutic protocol procedure on vertical ground reaction force values, obtained during two- and one-legged vertical hops?

Tengman et al. (2013) maintain that jumping ability, power, and muscle strength are important criteria that should be monitored before deciding whether to allow the patient–athlete to return to physical activity and training after lower limb injury treatment [42]. During dynamic physical activities, the operated AT must be able to withstand heavy loads during running, jumping, and drop jumps, so as not to be ruptured again. During drop jumps from high altitudes, the values of forces that act on the AT can reach the values of ten-fold the body weight [43]. Taking into account the possible loads exerted on the lower extremities, resulting from involvement in selected sports, we need to collect data on the gradual increase in load during the last phase of outpatient sports physiotherapy conducted and supervised by a physiotherapist [12]. Powell et al. (2018) recorded significantly lower values of kinematic parameters and vertical ground reaction force values during single-leg jumps six years after the surgical suturing of AT. The authors concluded that a higher risk of AT damage is involved in single-leg jumps rather than jogging [44].

Personalized postoperative templates of rehabilitation increase the repair strength of the AT [45]. The reference sources report methodological discrepancies in the applied physiotherapy protocols for patients after the surgical suturing of the AT [46,47,48]. Physiotherapeutic procedures conducted up to 4 months after surgical suturing of the AT have already been described [49,50]. The applied postoperative protocol in this research included the types and number of exercises, series, and rest breaks [19]. The types of comprehensive physiotherapeutic procedures applied at a given stage of the protocol are reported. Additionally, the protocol monitored the patients at different stages of the procedure and aimed to minimize recurrent injuries to the operated tendon [19]. Kadakia et al. (2009), in their postoperative protocol, performed standard and functional protocols using either plaster cast or orthosis [51]. This procedure considers basic recommendations, such as using orthosis, a gradual increase in mobility in the talocrural joint, using appropriate physiotherapeutic techniques, and exercising in water. Gruber et al. (2013) distinguished early and traditional postoperative physiotherapeutic protocols, including early physiotherapeutic procedures beginning within the first days after surgery [18]. Porter et al. (2014) determined the goals of each postoperative week without considering physiotherapeutic exercises [15]. Kadakia et al. (2009), who published their protocol for patients after the surgical suturing of AT, did not consider the number of exercises that were to be performed, or cooperation with a physician concerning the progress and supervision of the physiotherapy protocol [51]. Hutchison et al. (2015) maintained that the application of standardized postoperative procedures for patients after the surgical suturing of the AT may reduce the costs of health care and result in a quick return to the desired level of everyday physical activity [50]. Zellers et al. (2019) reported that, during the last stages of postoperative physiotherapy, attention should be focused on strengthening the medial head of the gastrocnemius muscle of the operated leg to improve jumping quality [52]. Bruman et al. (2014), in their review of the literature on physiotherapy protocols, focused mainly on the early loading and mobilization of the talocrural joint in the operated leg [46]. Zellers et al. (2019), based on different published postoperative physiotherapy protocols, concluded that early physiotherapy protocols should begin during the second postoperative week [47]. Carmont et al. (2020) suggested that the application of weightbearing does not influence the heel-rise height index but increases the AT resting phase one year after injury [53].

In recent years, studies have focused on the assessment of the correlation between the time and number of supervised 6 month physiotherapy protocols with more visits (x = 74) that have been reported compared with supervised physiotherapy with fewer visits (x = 32) [54]. The results indicate a favorable effect of supervised physiotherapy with more visits on the reduction in the differences in relative vertical ground reaction force values and LSI of vertical ground reaction force values between the operated and the uninvolved leg, compared with the values obtained in the control group. However, it is difficult to compare the results of the cited studies to this study’s results since the cited research was conducted in patients after the arthroscopic reconstruction of the anterior cruciate ligament (ACLR) [55]. Calculating the limb symmetry index is an easy approach for determining the level of symmetry or asymmetry in the assessed and monitored parameters reflecting jumping ability, biomechanical parameters of the lower limbs, or the studied values of biomechanical parameters obtained for specific muscle groups [56]. Myer et al. (2008) suggested that an LSI rate equal to or higher than 90 minimizes the risk of reinjury to the operated limb [57]. In this study, the number of operated ATs was similar in the dominant and nondominant lower limbs and amounted to 47.82%, meeting the required criteria established for an LSI ≥ 90 [58]. Unfortunately Group I did not meet these criteria, as opposed to Group II (control). Disorders of the symmetry of load exerted on the lower limbs and neuromuscular control in the long term can lead to degenerative changes in the ankle and knee joints and, consequently, to a deteriorated quality of life [59,60]. Brorsson et al. (2017) did not observe any improvement in the peak torque of the lower leg muscles during the two-year observation of patients after the surgical suturing of the AT, and these results remained unchanged even seven years after the surgery [61]. The introduction of jumping exercises during the final stages of physiotherapy [19] aims to minimize the differences between the values corresponding to strength, muscle power, and jumping ability in the operated leg and the uninvolved leg [62]. Schepull et al. (2007) state that after the surgical suturing of the AT, the final result may depend on patients’ motivation for exercise and participation in the physiotherapy program [63].

With reference to the previously mentioned results from patients after ACLR [22,54,55], supervised postoperative physiotherapy should be carried out within a longer postoperative period, which requires scientific evidence and, thus, further research.

The authors are aware that, due to the very strict selection of patients, the research ultimately included a small number of patients (23). Another limitation of this research is that the assessment of relative vertical ground reaction force values during two- and one-legged vertical hops was not completed in a sample of female patients. None of the female patients (*n* = 9) completed all five stages of supervised postoperative physiotherapy in the 6 months period; therefore, according to the exclusion criteria in the study of both men and women, they were excluded from the analysis. The authors are also aware that future research should include, for example, AT and ankle functional evaluation scales, such as the Achilles tendon Total Rupture Scale (ATRS) or the Ankle-Hindfoot score of American Orthopaedic Foot and Ankle Society (AOFAS); the assessment of strength in muscles affecting the talocrural joints; and the assessment and analysis of other types of locomotion related to the clinical assessment of patients after the surgical suturing of AT. In the future, the presented research should be performed using a larger sample and include female subjects.

The problem presented by the authors in the current study is important for patients–athletes who want to return to sports activity after the surgical suturing of the AT, as one of the most important criteria for this is to restore dynamic and symmetrical two- and one-legged vertical hops. Using a deep jump (DJ), counter-movement jump (CMJ), and squat jump (SJ) for research purposes, which are performed for two or three repetitions, may not reproduce actual conditions during future loads occurring in sports with a high frequency of hopping within a short time in situation like defense or attack in team sports as basketball, volleyball, and football. Vertical ankle hops simulate this condition, which is important in clinical application [13,26,27,28,30,44,61,64].

## 5. Conclusions

Our research showed that conducting an average of 42 visits of supervised postoperative physiotherapy for 6 months in patients after the surgical suturing of the Achilles tendon using an open Keesler’s technique was insufficient to obtain similar values of average relative vertical ground reaction forces and their LSI during two- and one-legged vertical hops.

The hypothesis, that a higher number of supervised physiotherapeutic visits performed on patients for six months after the surgical suturing of the Achilles tendon would result in higher values of the average relative vertical ground reaction forces during two- and one-legged vertical hops, was confirmed.

## Figures and Tables

**Table 1:** The between-group comparison of the values corresponding to body mass, body height, age, body mass index (BMI), the duration of postoperative physical therapy, and the number of postoperative physical therapy sessions.

Experimental Group	I (*n* = 23)	II (*n* = 23)	*p*
Age (years)	35.61 ± 8.99	31.87 ± 4.85	0.088
Body mass (kg)	87.57 ± 10.22	81.13 ± 11.27	0.019
Body height (cm)	184.65 ± 7.83	178.57 ± 5.58	0.004
BMI (kg∙m^−2^)	25.63 ± 1.82	25.36 ± 2.43	0.312
Duration of SVPPh (months)	6 months	n/a	–
Number of SVPPh sessions	42.22 ± 15.62	n/a	–
Operated leg	Right (*n* = 11), Left (*n* = 12)	Right (*n* = 0), Left (*n* = 0)	–
Dominant leg	Right (*n* = 21), Left (*n* = 2)	Right (*n* = 22), Left (*n* = 1)	–

Mean values—x; standard deviations—±; statistical significance level—*p*; patient’s body mass multiplied by patient’s body height in m^−2^—kg∙m^−2^; not applicable—n/a; supervised postoperative physiotherapy—SVPPh. Group I—after surgical suturing of Achilles tendon; Group II—control.

**Table 2:** Comparison of the between-group and intragroup values corresponding to pain; Thompson’s test results; 10 m unassisted walking; ankle joint and shin circumference; range of motion; full range of motion in sagittal plane, plantar flexion, and dorsiflexion, of the foot.

Tested Parameters	Group	Operated/Right Legs	Unoperated/Left Legs	*p*
Pain in VAS	I	0.00 ± 0.00	0.00 ± 0.00	-
	II	0.00 ± 0.00	0.00 ± 0.00	-
	*p*	–	–	–
Negative Thomson’s test results	I	23	23	–
	II	23	23	–
	*p*	–	–	–
10 m unassisted walking	I	23	23	–
	II	23	23	–
	*p*	–	–	–
Ankle circumference (cm)	I	27.66 ± 1.27	27.24 ± 1.39	*p* ≤ 0.001
	II	26.91 ± 1.50	26.91 ± 1.50	1.00
	*p*	0.061	0.303	
Shine circumference (cm)	I	38.38 ± 1.79	39.33 ± 1.56	*p* ≤ 0.001
	II	39.48 ± 5.20	39.26 ± 5.25	0.236
	*p*	0.574	0.103	
Full ROM of ankle joint in sagittal plane (°)	I	58.26 ± 9.69	59.43 ± 10.51	0.233
	II	61.89 ± 6.44	61.98 ± 6.40	0.747
	*p*	0.143	0.388	
DF ROM of ankle joint (°)	I	−14.13 ± 3.99	13.74 ± 8.14	0.572
	II	−14.02 ± 4.94	−14.37 ± 4.37	0.213
	*p*	0.935	0.697	
PF ROM of ankle joint (°)	I	44.13 ± 8.56	45.70 ± 6.64	0.273
	II	47.87 ± 6.03	47.61 ± 5.40	0.655
	*p*	0.067	0.160	

Mean values—x; standard deviations—±; statistical significance level—*p*; visual analogue scale—VAS; full range of motion of ankle joint in sagittal plane—Full ROM; plantar flexion range of motion of ankle joint—PF ROM; dorsiflexion range of motion of ankle joint—DF RO; Group I—after surgical suturing of Achilles tendon; Group II—control.

**Table 3:** The between-group comparative analysis using ANOVA and Tukey’s test to determine significant differences in the vertical ground reaction force component during two- and one-legged vertical hops for Group I and in the right and left legs for Group II.

**ANOVA**				
	**Operated Leg in** **Group I**	**Right Leg in Group II**	**Left Leg in Group II**	***p***
RvGRF–TLH (N·kg^−1^)	19.19 ± 5.03	28.95 ± 5.12	28.67 ± 4.94	*p* ≤ 0.001
RvGRF–OLH (N·kg^−1^)	26.50 ± 5.94	32.96 ± 5.38	33.60 ± 4.61	*p* ≤ 0.001
**TUKEY’S TEST**				
**Assessed Groups**	**Assessed Legs**	**RvGRF–OLH (N·kg^−1^)**	**RvGRF–TLH (N·kg^−1^)**	
Group I and II	Operated to Right	*p* ≤ 0.001	*p* ≤ 0.001	
Group I and II	Operated to Left	*p* ≤ 0.001	*p* ≤ 0.001	
Group II and II	Right to Left	*p* = 0.913	*p* = 0.981	

Mean values—x; standard deviations—±; difference significance level—*p*; relative vertical ground reaction forces component—RvGRF; Group I—after surgical suturing of Achilles tendon; Group II—control; two-legged vertical hops—TLH; one-legged vertical hops—OLH.

**Table 4:** Correlation between the number of postoperative physical therapy visits and the obtained values of the vertical component ground reaction forces for two- and one-legged vertical hops and their limb symmetry indices in Group I.

Test Correlation		RvGRF TLH Operated Leg	RvGRF TLH Unoperated Leg	RvGRF OLH Operated Leg	RvGRF OLH Unoperated Leg	LSI of vGRF TLH	LSI of vGRF OLH
Number of SVPPh visits	*r*	0.503	0.175	0.505	0.268	0.399	0.332
	*p*	0.014	0.424	0.014	0.217	0.059	0.122

Values of relative vertical ground reaction force—RvGRF; limb symmetry index—LSI; vertical ground reaction force—vGRF; the significance level of statistical differences—*p*; correlation coefficient *p* < 0.005—marked in bold line; two-legged vertical hops—TLH; one-legged vertical hops—OLH.

## Data Availability

This study was a purely retrospective and observational study. Results are available upon request to the editors and reviewers.

## References

[1] Malvankar S., Khan W. (2011). Evolution of the Achilles tendon: The athlete’s Achilles heel?. Foot.

[2] Maffulli N., Longo U.G., Maffulli G.D., Khanna A., Denaro V. (2011). Achilles Tendon Ruptures in Elite Athletes. Foot Ankle Int..

[3] Egger A.C., Berkowitz M.J. (2017). Achilles tendon injuries. Curr. Rev. Musculoskelet. Med..

[4] Maffulli N., Via A.G., Oliva F. (2017). Chronic Achilles Tendon Rupture. Open Orthop. J..

[5] Chianca V., Zappia M., Oliva F., Luca B., Maffulli N. (2020). Post-operative MRI and US appearance of the Achilles tendons. J. Ultrasound.

[6] Romero-Morales C., Martín-Llantino P.J., Calvo-Lobo C., Palomo-López P., López-López D., Pareja-Galeano H., Rodríguez-Sanz D. (2019). Comparison of the sonographic features of the Achilles Tendon complex in patients with and without achilles tendinopathy: A case–control study. Phys. Ther. Sport.

[7] Tarantino D., Palermi S., Sirico F., Corrado B. (2020). Achilles Tendon Rupture: Mechanisms of Injury, Principles of Rehabilitation and Return to Play. J. Funct. Morphol. Kinesiol..

[8] Oliva F., Rugiero C., Via A.G., Baldassarri M., Bernardi G., Biz C., Bossa M., Buda R., Buonocore D., Chianca V. (2019). IS Mu. LT Achilles tendon ruptures guidelines. Muscle Ligaments Tendons J..

[9] Lantto I., Heikkinen J., Flinkkila T., Ohtonen P., Siira P., Laine V., Leppilahti J. (2016). A Prospective Randomized Trial Comparing Surgical and Nonsurgical Treatments of Acute Achilles Tendon Ruptures. Am. J. Sports Med..

[10] Maffulli G., Del Buono A., Richards P., Oliva F., Maffulli N. (2017). Conservative, minimally invasive and open surgical repair for management of acute ruptures of the Achilles tendon: A clinical and functional retrospective study. Muscle Ligaments Tendons J..

[11] Maffulli N., D’Addona A., Maffulli G.D., Gougoulias N., Oliva F. (2020). Delayed (14–30 Days) Percutaneous Repair of Achilles Tendon Ruptures Offers Equally Good Results as Compared with Acute Repair. Am. J. Sports Med..

[12] Park S.-H., Lee H.S., Young K.W., Seo S.G. (2020). Treatment of Acute Achilles Tendon Rupture. Clin. Orthop. Surg..

[13] Tanaka R., Imaya T., Katsuki S., Sanada T., Fukai A., Honda E., Yoshitomi H. (2021). Factors Associated to Return to Sport after Surgical Repair of Achilles Tendon Ruptures. A Clinical and Functional Retrospective Study. Muscle Ligaments Tendons J..

[14] Yang X., Meng H., Quan Q., Peng J., Lu S., Wang A. (2018). Management of acute Achilles tendon ruptures. Bone Jt. Res..

[15] Porter M.D., Shadbolt B. (2014). Randomized controlled trial of accelerated rehabilitation versus standard protocol following surgical repair of ruptured Achilles tendon. ANZ J. Surg..

[16] Zhao J.-G., Meng X.-H., Liu L., Zeng X.-T., Kan S.-L. (2017). Early functional rehabilitation versus traditional immobilization for surgical Achilles tendon repair after acute rupture: A systematic review of overlapping meta-analyses. Sci. Rep..

[17] Gould H.P., Bano J.M., Akman J.L., Fillar A.L. (2021). Postoperative Rehabilitation Following Achilles Tendon Repair: A Systematic Review. Sports Med. Arthrosc. Rev..

[18] Gruber J., Giza E., Zachazewski J., Mandelbaum B.R., Maxey L., Magnusson J. (2013). Achilles Tendon Repair and Rehabilitation. Rehabilitation for the Postsurgical Orthopedic Patient.

[19] Czamara A. (2007). Physiotherapeutic Treatments after Surgery of Total Achilles Tendon Rupture. J. Orthop. Trauma Surg. Relat. Res..

[20] Schenck R.C., Blaschak M., Lance E.D., Turturro T.C., Holmes C.F. (1997). A prospective outcome study of rehabilitation programs and anterior cruciate ligament reconstruction. Arthrosc. J. Arthrosc. Relat. Surg..

[21] Huang J., Wang C., Ma X., Wang X., Zhang C., Chen L. (2014). Rehabilitation Regimen After Surgical Treatment of Acute Achilles Tendon Ruptures: A systematic review with meta-analysi. Am. J. Sports Med..

[22] Królikowska A., Sikorski Ł., Czamara A., Reichert P. (2018). Effects of Postoperative Physiotherapy Supervision Duration on Clinical Outcome, Speed, and Agility in Males 8 Months After Anterior Cruciate Ligament Reconstruction. Med. Sci. Monit..

[23] Ebert J.R., Edwards P., Yi L., Joss B., Ackland T., Carey-Smith R., Buelow J.-U., Hewitt B. (2017). Strength and functional symmetry is associated with post-operative rehabilitation in patients following anterior cruciate ligament reconstruction. Knee Surg. Sports Traumatol. Arthrosc..

[24] Glazebrook M., Rubinger D. (2019). Functional Rehabilitation for Nonsurgical Treatment of Acute Achilles Tendon Rupture. Foot Ankle Clin..

[25] Dams O.C., Akker-Scheek I.V.D., Diercks R.L., Wendt K.W., Bosma E., Van Raaij T.M., Munzebrock A.V., Zijlstra W.P., Zwerver J., Reininga I.H.F. (2019). The recovery after Achilles tendon rupture: A protocol for a multicenter prospective cohort study. BMC Musculoskelet. Disord..

[26] Zellers J.A., Carmont M.R., Silbernagel K.G. (2016). Return to play post-Achilles tendon rupture: A systematic review and meta-analysis of rate and measures of return to play. Br. J. Sports Med..

[27] Olsson N., Nilsson-Helander K., Karlsson J., Eriksson B.I., Thomée R., Faxén E., Silbernagel K.G. (2011). Major functional deficits persist 2 years after acute Achilles tendon rupture. Knee Surg. Sports Traumatol. Arthrosc..

[28] Willy R.W., Brorsson A., Powell H.C., Willson J., Tranberg R., Silbernagel K.G. (2017). Elevated Knee Joint Kinetics and Reduced Ankle Kinetics Are Present During Jogging and Hopping After Achilles Tendon Ruptures. Am. J. Sports Med..

[29] Jandacka D., Plesek J., Skypala J., Uchytil J., Silvernail J.F., Hamill J. (2018). Knee Joint Kinematics and Kinetics During Walking and Running After Surgical Achilles Tendon Repair. Orthop. J. Sports Med..

[30] Nilsson-Helander K., Silbernagel K.G., Thomeé R., Faxén E., Olsson N., Eriksson B.I., Karlsson J. (2010). Acute Achilles Tendon Rupture. Am. J. Sports Med..

[31] Wearing S.C., Kuhn L., Pohl T., Horstmann T., Brauner T. (2020). Transmission-Mode Ultrasound for Monitoring the Instantaneous Elastic Modulus of the Achilles Tendon During Unilateral Submaximal Vertical Hopping. Front. Physiol..

[32] Van Meer B.L., Meuffels D.E., Reijman M. (2017). A Comparison of the Standardized Rating Forms for Evaluation of Anterior Cruciate Ligament Injured or Reconstructed Patients. The Anterior Cruciate Ligament: Reconstruction and Basic Science.

[33] Henríquez H., Muñoz R., Carcuro G., Bastías C. (2012). Is Percutaneous Repair Better Than Open Repair in Acute Achilles Tendon Rupture?. Clin. Orthop. Relat. Res..

[34] Czamara A. (2011). Biomechanical assessment of unilateral and bilateral landing symmetry during rehabilitation following anterior cruciate ligament reconstruction (ACLR). Pol. J. Sports Med..

[35] Baechle T.R., Earle R.W. (2008). Essentials of Strength Training and Conditioning.

[36] Królikowska A., Czamara A., Szuba Ł., Reichert P. (2018). The Effect of Longer versus Shorter Duration of Supervised Physiotherapy after ACL Reconstruction on the Vertical Jump Landing Limb Symmetry. BioMed Res. Int..

[37] Matheson A.L., Duffy S., Maroof A., Gibbons R., Duffy C., Roth J. (2013). Intra- and inter-rater reliability of jumping mechanography muscle function assessments. J. Musculoskelet Neuronal Interact..

[38] Sikorski Ł., Czamara A. (2021). Assessment of Effectiveness of 15 Weeks of Physical Therapy on Biplanar Ankle Mobility, Gait and Pain Level in Patients Following Operative Repair of the Achilles Tendon. Ortop. Traumatol. Rehabil..

[39] Kuo A.D. (2007). The six determinants of gait and the inverted pendulum analogy: A dynamic walking perspective. Hum. Mov. Sci..

[40] Somford M.P., Hoornenborg D., Wiegerinck J.I., Weme R.A.N. (2016). Are You Positive That the Simmonds-Thompson Test Is Negative? A Historical and Biographical Review. J. Foot Ankle Surg..

[41] Mukaka M.M. (2012). Statistics corner: A guide to the appropriate use of correlation coefficient in medical research. Malawi Med. J..

[42] Tengman T., Riad J. (2013). Three-Dimensional Gait Analysis Following Achilles Tendon Rupture with Nonsurgical Treatment Reveals Long-Term Deficiencies in Muscle Strength and Function. Orthop. J. Sports Med..

[43] Burdett R.G. (1982). Forces predicted at the ankle during running. Med. Sci. Sports Exerc..

[44] Powell H.C., Silbernagel K.G., Brorsson A., Tranberg R., Willy R. (2018). Individuals Post Achilles Tendon Rupture Exhibit Asymmetrical Knee and Ankle Kinetics and Loading Rates During a Drop Countermovement Jump. J. Orthop. Sports Phys. Ther..

[45] Thomopoulos S., Parks W.C., Rifkin D.B., Derwin K.A. (2015). Mechanisms of tendon injury and repair. J. Orthop. Res..

[46] Brumann M., Baumbach S., Mutschler W., Polzer H. (2014). Accelerated rehabilitation following Achilles tendon repair after acute rupture—Development of an evidence-based treatment protocol. Injury.

[47] Zellers J.A., Christensen M., Kjær I.L., Rathleff M.S., Silbernagel K.G. (2019). Defining Components of Early Functional Rehabilitation for Acute Achilles Tendon Rupture: A Systematic Review. Orthop. J. Sports Med..

[48] Mark-Christensen T., Troelsen A., Kallemose T., Barfod K.W. (2014). Functional rehabilitation of patients with acute Achilles tendon rupture: A meta-analysis of current evidence. Knee Surg. Sports Traumatol. Arthrosc..

[49] Ryu C.H., Lee H.S., Seo S.G., Kim H.Y. (2018). Results of tenorrhaphy with early rehabilitation for acute tear of Achilles tendon. J. Orthop. Surg..

[50] Hutchison A.M., Topliss C., Beard D., Evans R.M., Williams P. (2015). The treatment of a rupture of the Achilles tendon using a dedicated management programme. Bone Jt. J..

[51] Kadakia A.R., Short K., Myerson M.S. (2008). Rehabilitation After Acute Ruptures of the Achilles Tendon. The Achilles Tendon: Treatment and Rehabilitation.

[52] Zellers J.A., Marmon A.R., Ebrahimi A., Silbernagel K.G. (2019). Lower extremity work along with triceps surae structure and activation is altered with jumping after Achilles tendon repair. J. Orthop. Res..

[53] Carmont M.R., Brorsson A., Karlsson J., Nilsson-Helander K. (2020). No difference in Achilles Tendon Resting Angle, Patient-reported outcome or Heel-rise height Index between Non- and Early weightbearing the First Year after an Achilles Tendon Rupture. Muscle Ligaments Tendons J..

[54] Czamara A., Krzemińska K., Widuchowski W., Dragan S.L. (2021). The Muscle Strength of the Knee Joint after ACL Reconstruction Depends on the Number and Frequency of Supervised Physiotherapy Visits. Int. J. Environ. Res. Public Health.

[55] Królikowska A., Czamara A., Reichert P. (2018). Between-Limb Symmetry during Double-Leg Vertical Hop Landing in Males an Average of Two Years after ACL Reconstruction is Highly Correlated with Postoperative Physiotherapy Supervision Duration. Appl. Sci..

[56] Zwolski C., Schmitt L.C., Thomas S., Hewett T.E., Paterno M.V. (2016). The Utility of Limb Symmetry Indices in Return-to-Sport Assessment in Patients with Bilateral Anterior Cruciate Ligament Reconstruction. Am. J. Sports Med..

[57] Myer G.D., Paterno M.V., Ford K., Hewett E.T. (2008). Neuromuscular Training Techniques to Target Deficits Before Return to Sport After Anterior Cruciate Ligament Reconstruction. J. Strength Cond. Res..

[58] Souissi S., Chaouachi A., Burnett A., Hue O., Bouhlel E., Chtara M., Chamari K. (2020). Leg asymmetry and muscle function recovery after anterior cruciate ligament reconstruction in elite athletes: A pilot study on slower recovery of the dominant leg. Biol. Sport.

[59] Markström J.L., Tengman E., Häger C.K. (2017). ACL-reconstructed and ACL-deficient individuals show differentiated trunk, hip, and knee kinematics during vertical hops more than 20 years post-injury. Knee Surg. Sports Traumatol. Arthrosc..

[60] Thomas A.C., Hubbard-Turner T., Wikstrom E., Palmieri-Smith R.M. (2017). Epidemiology of Posttraumatic Osteoarthritis. J. Athl. Train..

[61] Brorsson A., Silbernagel K.G., Olsson N., Helander K.N. (2017). Calf Muscle Performance Deficits Remain 7 Years After an Achilles Tendon Rupture. Am. J. Sports Med..

[62] Markovic G., Mikulic P. (2010). Neuro-Musculoskeletal and Performance Adaptations to Lower-Extremity Plyometric Training. Sports Med..

[63] Schepull T., Kvist J., Andersson C., Aspenberg P. (2007). Mechanical properties during healing of Achilles tendon ruptures to predict final outcome: A pilot Roentgen stereophotogrammetric analysis in 10 patients. BMC Musculoskelet. Disord..

[64] Lamontagne M., Kennedy M.J. (2013). The Biomechanics of Vertical Hopping: A Review. Res. Sports Med..

